# Sex differences in the association between composite dietary antioxidant index and hyperlipidemia: Insights from NHANES

**DOI:** 10.1371/journal.pone.0316130

**Published:** 2025-01-10

**Authors:** Xiaofan Miao, Bixia Li, Zhixian Zhu, Tao Yang

**Affiliations:** 1 Department of Cardiology, The Affiliated Hospital of Nanjing University of Chinese Medicine, Nanjing, China; 2 Clinical Lab, The Affiliated Hospital of Nanjing University of Chinese Medicine, Nanjing, China; 3 School of Artificial Intelligence and Information Technology, Nanjing University of Chinese Medicine, Nanjing, China; King Abdulaziz University Faculty of Medicine, SAUDI ARABIA

## Abstract

**Background:**

Previous studies have shown that both the composite dietary antioxidant index (CDAI) and sex are strongly associated with a variety of cardiovascular diseases, but sex differences between CDAI and hyperlipidemia are unknown.

**Objective:**

This study utilized data from the National Health and Nutrition Examination Survey (NHANES) to investigate the sex differences between CDAI and hyperlipidemia.

**Method:**

We calculated the CDAI of the six dietary antioxidants using data from NHANES, explored the relationship between CDAI and the prevalence of hyperlipidemia using multivariate logistic regression analysis, and analyzed for potential nonlinear associations using restricted cubic spline. Finally, the association between CDAI and hyperlipidemia was further explored using multivariate logistic regression in different genders.

**Results:**

The study included a total of 34,754 participants with a mean age of 47.04 years, of whom 49.37% were man. In a fully adjusted multivariable binary logistic regression model, CDAI was negatively associated with the prevalence of hyperlipidemia (OR = 0.99, 95% CI:0.98–0.99). In addition, participants in the highest quartile had a lower risk of hyperlipidaemia compared with the lowest quartile of CDAI (OR = 0.83, 95%CI: 0.76–0.92). We also found a non-linear relationship (non-linear P = 0.003, Inflection point = -0.179). Finally, we found that the association between CDAI and the prevalence of hyperlipidemia was significantly stronger in the female population than in the male population (P for interaction <0.05).

**Conclusion:**

Our study highlights the L-shaped association between CDAI and the prevalence of hyperlipidemia in the general adult population. In addition, this association was more significant in the female population than in the male population.

## Introduction

In the past few decades, hyperlipidemia has received widespread attention as a common metabolic disorder. Hyperlipidemia is characterized by elevated levels of lipids in the blood, particularly cholesterol and triglycerides above the normal range [1]. It is associated with various diseases, such as coronary heart disease, stroke, and other cardiovascular diseases (CVD) [2,3]. Individuals with hyperlipidemia have twice the risk of developing CVD compared to those with normal lipid levels [4]. A study conducted in the Dutch population found that elevated levels of low-density lipoprotein (LDL) are associated with an increased risk of CVD [5]. Additionally, another study indicated that lowering triglyceride levels in the blood can significantly reduce the risk of developing and dying from coronary heart disease [6]. Gender disparities significantly influence the incidence of various diseases. For instance, certain malignancies, such as bladder and esophageal cancers, exhibit a higher prevalence in males compared to females. Conversely, autoimmune diseases like Hashimoto’s thyroiditis and systemic lupus erythematosus are more prevalent in females [7–10]. Gender also plays a crucial role in CVD [11,12]. Research indicates that the onset of cardiovascular disease occurs approximately 5–10 years earlier in women than in men, and women tend to have a poorer prognosis. For instance, the risk of mortality due to diabetes-induced myocardial infarction is 50% higher in women than in men [13]. In the context of contemporary diets high in calories and sedentary lifestyles, the rates of hyperlipidemia-related morbidity and mortality remain elevated [14,15]. While numerous studies have documented the prevalence of CVD in both genders, research examining the impact of gender on the progression of hyperlipidemia is relatively sparse [16].

Numerous studies have highlighted the significant association between diet and hyperlipidemia. The nutrients and constituents present in a balanced diet can impact cholesterol metabolism and blood lipid profiles, thereby influencing the risk of hyperlipidemia [17]. Research has shown that the consumption of saturated fatty acids and trans fatty acids in the diet is closely correlated with the risk of hyperlipidemia [18–20]. Moreover, dietary components such as fiber, polysaccharides, omega-3 fatty acids, and plant sterols have been found to lower blood lipid levels and aid in the prevention of hyperlipidemia [21–25]. Furthermore, gender differences are evident in dietary habits. Males typically consume diets that are higher in animal protein, whereas females’ diets often include greater amounts of fat [26]. However, the relationship between overall dietary patterns and the risk of hyperlipidemia across genders has not been comprehensively explored in the current study.

The composite dietary antioxidant index (CDAI) serves as a comprehensive measure to evaluate the antioxidant content and capacity of antioxidants in the diet, encompassing vitamins A, C, and E, as well as minerals such as magnesium, selenium, and zinc. Prior research has established a close relationship between CDAI and various cardiovascular conditions, including hypertension, heart failure, coronary heart disease, stroke, and hyperlipidemia [27–31]. However, men and women differ in their cardiovascular risk trajectories **[32]**. The specific association of sex differences between CDAI and hyperlipidemia remains uncertain.

By analysing the National Health and Nutrition Examination Survey (NHANES) database, the main aim of this study was to explore sex differences between CDAI and hyperlipidemia, with a view to providing more targeted dietary intervention strategies for the management of hyperlipidemia in sex-specific populations.

## Methods

### Study design and population

**Fig 1:**
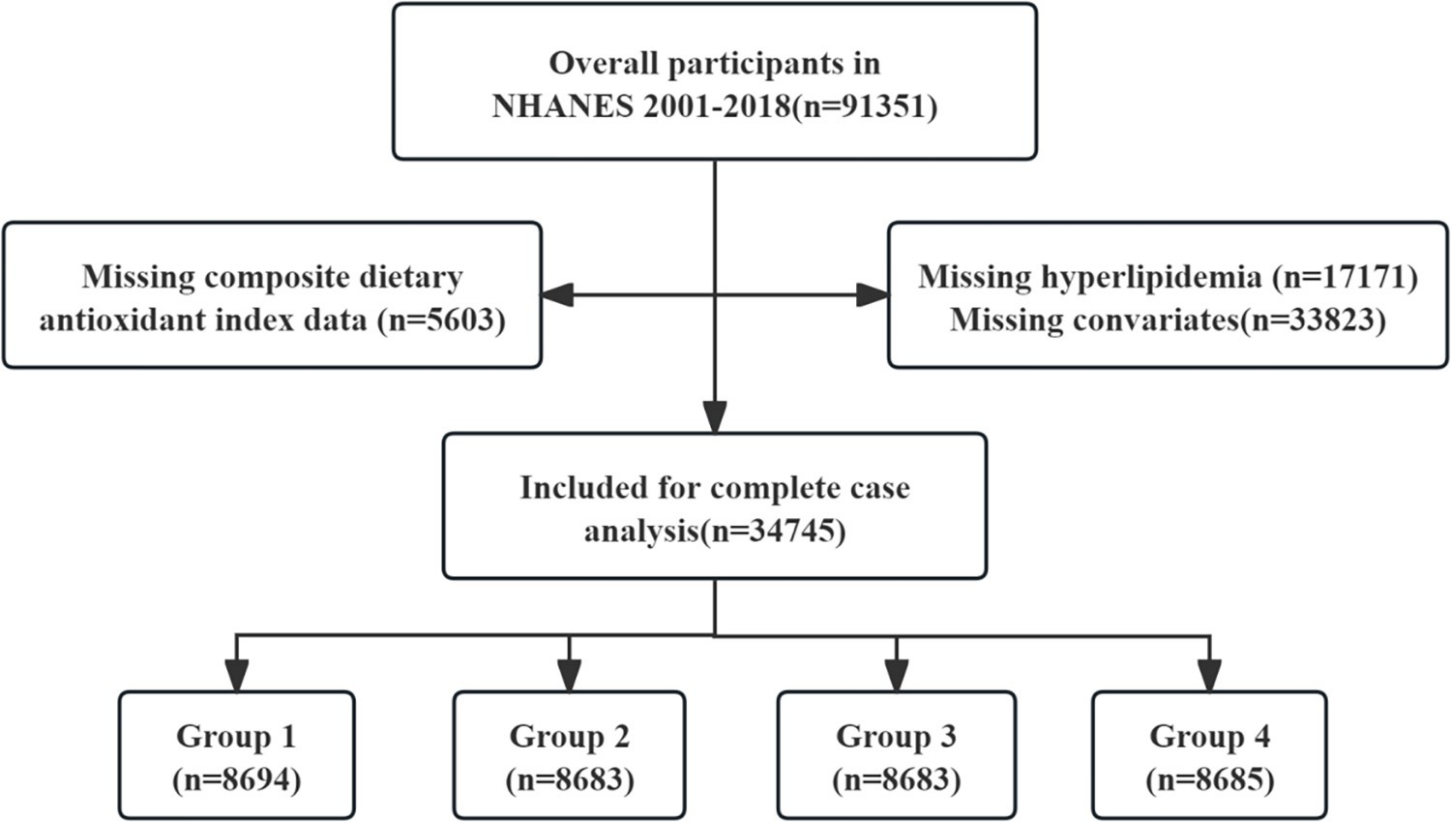
Flow chart of the study subjects.

The NHANES is a nationally representative survey conducted by the National Center for Health Statistics (NCHS), a division of the Centers for Disease Control. Data were collected through interviews and physical examinations. The study used stratified multistage probability sampling to draw participants from the general population nationwide to ensure representative results. The study followed the ethical guidelines of the Declaration of Helsinki for human research, the NHANES research protocol was approved by the NCHS Research Ethics Review (IBR) Board (survey name: NHANES 2021–2022, NCHS IRB/ERB protocol number or description: protocol #2021–05), and informed written consent was obtained from all participants. The datasets utilized in the current study are available on the NHANES website (https://www.cdc.gov/nchs/nhanes/index.htm). Nine cycles of NHANES data spanning from 2001 to 2018 were used. Each cycle of data comprises demographic information, dietary records, physical examination results, laboratory findings, and questionnaire responses. A total of 91,351 participants were investigated in this study, of which 5,603 were excluded due to missing CDAI data, 17,171 due to missing hyperlipidaemia data, and 33,823 due to missing information on other covariates, and then a total of 34,745 were enrolled in the study, and the participants were classified into four groups based on quartiles of the CDAI values, as shown in Fig 1.

### Exposure variables and outcome variable

In this study, CDAI was the exposure variable. Using the NHANES computer-assisted dietary interview system, researchers collected and recorded the types and amounts of food and beverages consumed by participants over a 24-hour period (excluding any nutrients from dietary supplements or medications). Antioxidant intake was then determined by querying the USDA’s Dietary Research Food and Nutrient Database. The calculation method for CDAI aligns with the approach outlined by Wright et al [33]. Six key dietary vitamins and minerals, specifically vitamins A, C, E, selenium, zinc, and carotenoids, were standardized by subtracting the population mean and dividing by the standard deviation. The specific calculation method is as follows:

$$CDAI=\sum_{i=1}^{n=6}\frac{Individual\;Intake-Mean}{SD}$$

In this study, hyperlipidemia is considered as the outcome variable. The Adult Treatment Panel III guidelines of the National Cholesterol Education Program define hyperlipidemia as meeting the following criteria: a total cholesterol level of 200mg/dL or above, triglyceride levels of 150mg/dL or higher, male high-density lipoprotein (HDL) levels below 40mg/dL, female HDL levels below 50mg/dL, or LDL levels at or above 130mg/dL [34]. Furthermore, individuals who are reported to be taking cholesterol-lowering medications are also categorized as having hyperlipidemia.

### Covariates

Based on previous research and clinical experience, we have identified a set of covariates that may impact the relationship between CDAI and hyperlipidemia. The continuous variables considered in this study are age, body mass index (BMI), and poverty income ratio (PIR). Categorical variables include sex, race (categorized as Mexican American, Non-Hispanic Black, Non-Hispanic White, Other Hispanic, and Other Race), marital status (married, never married, separated), education level (less than high school, high school, college or above), smoking (never, former, now), drinking (yes, no), CVD, chronic kidney disease (CKD) and type 2 diabetes mellitus (DM) [35–37]. CVD was diagnosed based on self-reported doctor diagnoses of congestive heart failure, coronary heart disease, myocardial infarction, or stroke. CKD is defined as an estimated glomerular filtration rate < 60 ml/min or urinary albumin-to-creatinine ratio ≥ 30 mg/g. Type 2 DM diagnosed based on self-report, HbA1c levels > 6.5%, or fasting plasma glucose levels > 126 mg/dL, and hypertension defined as average systolic pressure ≥ 140 mmHg, diastolic pressure ≥ 90 mmHg, or current use of hypertension medication.

### Statistical analysis

Participants in the study were categorized into four groups based on quartiles of the CDAI as follows: Q1: <-2.16, Q2: -2.16 to -0.18, Q3: -0.18 to 2.34, and Q4: ≥2.34. Continuous variables were presented as means and standard error, while categorical variables were shown as frequencies and percentages. To compare the baseline characteristics of the CDAI quartiles, a one-way analysis of variance was used for continuous variables and the chi-square test for categorical variables.

We used multivariate logistic regression analysis to control for potential confounders because our study was designed to explore the association between the CDAI and hyperlipidemia, taking into account the fact that multiple variables may have an impact on this association. In addition, logistic regression was applicable to binary outcome variables and was able to provide odds ratios (ORs) and 95% confidence intervals (CIs), facilitating the assessment of the strength and direction of the association. The Crude model was unadjusted, Model 1 was adjusted for age, sex, race, and PIR, and Model 2 additionally adjusted for marital status, education level, BMI, smoking, drinking, type 2 DM, hypertension, CKD, and CVD based on Model 1. The lowest quartile of CDAI was utilized as the reference when treated as a categorical variable in all three models. CDAI was also analyzed as a continuous variable using restricted cubic splines (RCS). We chose the RCS to analyze the nonlinear relationship between CDAI and hyperlipidemia because of its flexibility in modeling complex relationships between variables and its ability to identify potential inflection points, and to calculate the inflection point in the relationship between CDAI and hyperlipidemia if a nonlinear correlation between the two is found. Furthermore, hyperlipidemia patients were stratified into different sex subgroups. Interaction tests were conducted to examine the associations between CDAI and hyperlipidemia in male and female populations. ORs and 95% CIs were calculated for each analysis.

All statistical analyses were performed using R software (version 4.1.3) and processed using complex sampling by official recommendations, with a two-sided P value of less than 0.05 considered statistically significant.

## Results

### Baseline characteristics of study participants

Table 1 displays the baseline characteristics of the study participants. The average age of the subjects was 47.04 years, and males accounted for 49.37% of the population. The participants were divided into four groups according to the quartiles of the CDAI, statistical significance was observed across the four groups for various factors including age, sex, race, PIR, marital status, education level, BMI, smoking, drinking, CVD, CKD, hypertension, type 2 DM, and hyperlipidemia (P < 0.05). Participants with higher CDAI were characterized by being younger, having a higher proportion of males, higher PIR, and lower BMI. Additionally, this groups exhibited a higher percentage of non-Hispanic white individuals, married, those with a college education or above, drinking, and never smokers, and former smokers. They also had a lower prevalence of CVD, CKD, hypertension, type 2 DM, and hyperlipidemia compared to the lower quartiles of CDAI.

**Table 1:** Characteristics of the study population by CDAI quartiles.

	Total	Q1	Q2	Q3	Q4	P value
	(n = 34745)	(n = 8694)	(n = 8683)	(n = 8683)	(n = 8685)	
Age, years	47.04(0.21)	47.47(0.28)	47.57(0.25)	47.44(0.30)	45.83(0.30)	< 0.001
Sex (%)						< 0.001
Female	17240(50.63)	4645(56.60)	4228(50.47)	4222(49.58)	4145(46.96)	
Male	17505(49.37)	4049(43.40)	4455(49.53)	4461(50.42)	4540(53.04)	
Race (%)						< 0.001
Mexican American	5682(7.75)	1397(7.57)	1462(8.04)	1433(7.63)	1390(7.77)	
Non-Hispanic Black	6973(10.21)	2156(13.99)	1676(9.95)	1530(8.57)	1611(8.99)	
Non-Hispanic White	16417(71.00)	3745(66.54)	4161(71.16)	4305(73.20)	4206(72.33)	
Other Hispanic	2740(4.86)	740(5.64)	680(4.80)	683(4.64)	637(4.49)	
Other Race	2933(6.18)	656(6.27)	704(6.04)	732(5.97)	841(6.43)	
Marital status (%)						< 0.001
Married	18415(57.05)	4137(50.45)	4725(57.98)	4818(59.68)	4735(58.98)	
Never married	8623(24.84)	2246(27.02)	2043(24.13)	2039(22.75)	2295(25.74)	
Separated	7707(18.11)	2311(22.52)	1915(17.88)	1826(17.56)	1655(15.27)	
Education level (%)						< 0.001
Less than high school	1169(1.76)	364(2.37)	316(1.94)	282(1.60)	207(1.26)	
High School	15340(37.09)	4676(47.87)	3960(38.75)	3471(33.12)	3233(30.79)	
College or above	18236(61.15)	3654(49.77)	4407(59.32)	4930(65.29)	5245(67.95)	
BMI, kg/m^2^	28.89(0.07)	29.05(0.11)	29.08(0.10)	28.85(0.10)	28.64(0.13)	0.010
PIR	3.07(0.03)	2.67(0.03)	3.00(0.03)	3.24(0.03)	3.29(0.04)	< 0.001
CVD (%)						<0.001
No	30855(91.57)	7406(88.66)	7645(90.91)	7827(92.29)	7977(93.82)	
Yes	3890(8.43)	1288(11.34)	1038(9.09)	856(7.71)	708(6.18)	
Hyperlipidemia (%)						<0.001
No	9744(29.20)	2235(26.18)	2326(28.56)	2452(29.48)	2731(31.96)	
Yes	25001(70.80)	6459(73.82)	6357(71.44)	6231(70.52)	5954(68.04)	
CKD (%)						<0.001
No	28284(85.89)	6685(82.13)	6996(85.38)	7190(87.00)	7413(88.32)	
Yes	6461(14.11)	2009(17.87)	1687(14.62)	1493(13.00)	1272(11.68)	
Smoking (%)						<0.001
Never	18550(53.69)	4253(47.97)	4578(53.23)	4775(55.45)	4944(57.01)	
Former	8816(25.14)	2080(22.35)	2305(25.33)	2269(26.32)	2162(26.07)	
Now	7379(21.18)	2361(29.69)	1800(21.45)	1639(18.23)	1579(16.92)	
Drinking (%)						<0.001
No	10728(24.73)	3232(30.14)	2729(25.66)	2503(23.08)	2264(21.16)	
Yes	24017(75.27)	5462(69.86)	5954(74.34)	6180(76.92)	6421(78.84)	
Type 2 DM (%)						<0.001
No	28615(87.00)	6914(85.06)	7041(86.22)	7252(87.59)	7408(88.68)	
Yes	6130(13.00)	1780(14.94)	1642(13.78)	1431(12.41)	1277(11.32)	
Hypertension (%)						<0.001
No	19916(62.77)	4591(59.75)	4869(61.73)	5129(63.77)	5327(65.15)	
Yes	14829(37.23)	4103(40.25)	3814(38.27)	3554(36.23)	3358(34.85)	

Abbreviations: BMI, body mass index; PIR, poverty income ratio; DM, diabetes mellitus; CKD, chronic kidney disease; CVD, cardiovascular disease.

All values are expressed as a proportion (%) or mean ± standard error.

### The relationship between CDAI and hyperlipidemia

Table 2 explores the association between CDAI and hyperlipidemia using logistic regression model. When CDAI was considered as a continuous variable, the Crude model revealed a significant association between CDAI and hyperlipidemia, for every 1-unit increase in CDAI, the risk of hyperlipidemia decreased by 3% (OR = 0.97, 95% CI: 0.97–0.98). Following adjustments for confounding factors in Model 2, a significant association between CDAI and hyperlipidemia also persisted (OR = 0.99, 95%CI: 0.98–0.99). Furthermore, when CDAI was categorized into quartiles, in Model 2 adjusting for relevant variables, the adjusted OR and its 95%CI for CDAI from lowest to highest quartiles were as follows: 1.00, 0.88 (95%CI: 0.80–0.96), 0.86 (95%CI: 0.78–0.96), and 0.83 (95%CI: 0.76–0.92), participants in Q4 had a 17% lower risk of developing hyperlipidemia compared to those in Q1. Hyperlipidemia decreased with increased CDAI (P for trend < 0.001).

**Table 2:** Association between CDAI and hyperlipidemia in the general population.

	Crude model		Model 1		Model 2	
	OR (95%CI)	P	OR (95%CI)	P	OR (95%CI)	P
CDAI	0.97(0.97,0.98)	<0.001	0.98(0.97,0.99)	<0.001	0.99(0.98,0.99)	<0.001
CDAI quartiles						
Q1	ref		ref		ref	
Q2	0.89(0.81,0.97)	0.009	0.86(0.79,0.94)	0.002	0.88(0.80,0.96)	0.008
Q3	0.85(0.77,0.94)	0.001	0.82(0.74,0.91)	<0.001	0.86(0.78,0.96)	0.007
Q4	0.76(0.69,0.83)	<0.001	0.77(0.70,0.85)	<0.001	0.83(0.76,0.92)	<0.001
P for trend		<0.001		<0.001		<0.001

Crude model: No adjusted.

Model 1: Adjusted for age, sex, race, and PIR.

Model 2: Adjusted for age, sex, race, smoking, drinking, education, type 2 DM, hypertension, marital status, PIR, BMI, CKD, and CVD.

Abbreviations: BMI, body mass index; PIR, poverty income ratio; DM, diabetes mellitus; CKD, chronic kidney disease; CVD, cardiovascular disease.

### Dose-response association of CDAI and hyperlipidemia

**Fig 2:**
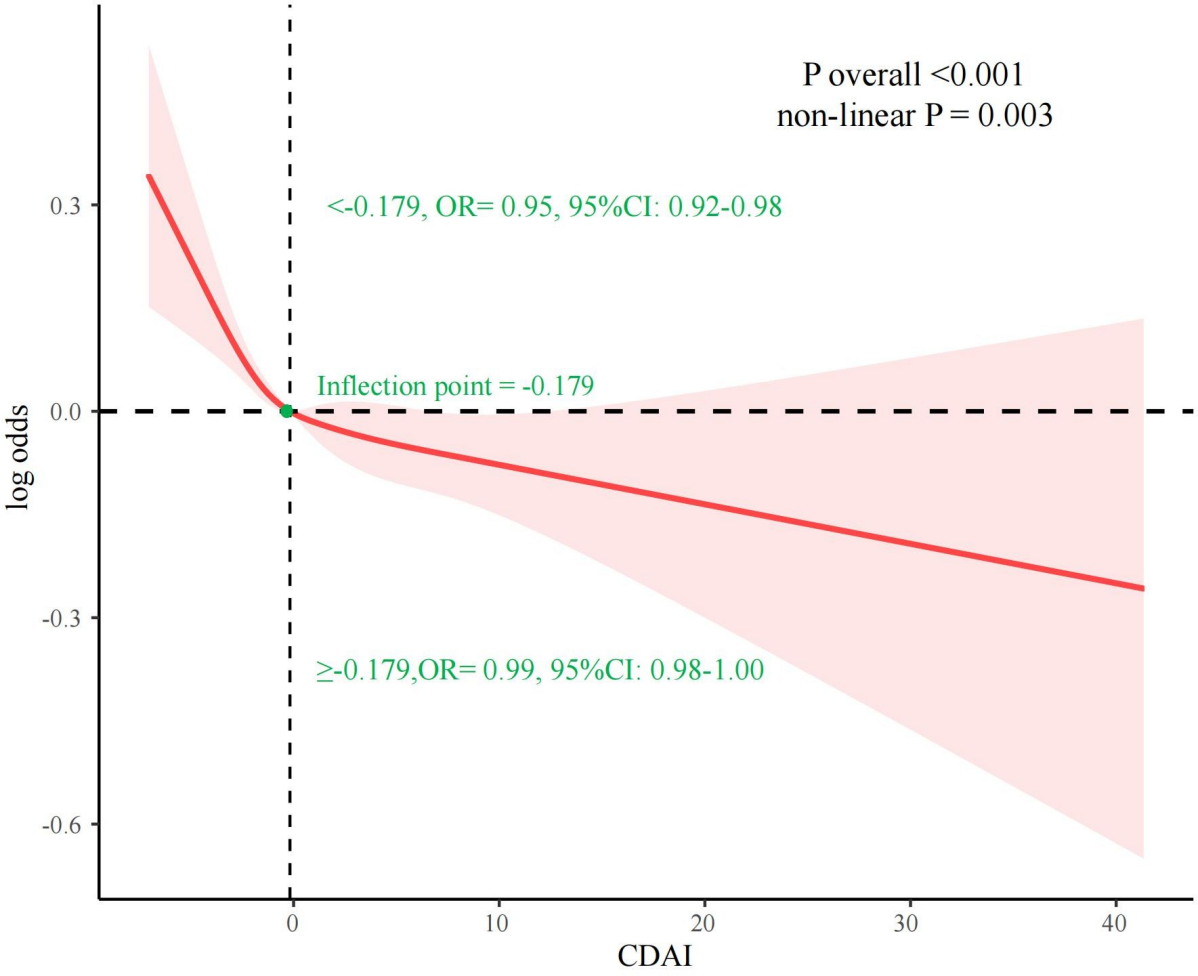
Restricted cubic spline (RCS) regression analysis of CDAI index and prevalence of hyperlipidemia.

Logistic regression results showed that CDAI was negatively associated with hyperlipidemia, and Fig 2 further applies RCS curves to assess the dose-response relationship between CDAI and hyperlipidemia. Upon adjusting for various confounding factors such as age, sex, race, PIR, education level, marital status, BMI, drinking, smoking, type 2 DM, hypertension, CKD, and CVD, the statistical significance of the association between CDAI and hyperlipidemia remained (overall P < 0.001). Furthermore, the analysis indicated a non-linear relationship between CDAI and hyperlipidemia (non-linear P = 0.003, Inflection point = -0.179). Before the inflection point, the prevalence of hyperlipidemia decreased significantly as the CDAI increased (OR = 0.95, 95% CI: 0.92–0.98), whereas after the inflection point, the trend of decreasing prevalence leveled off (OR = 0.99, 95% CI: 0.97–1.00).

### Sex differences in the association of CDAI and hyperlipidemia

Table 3 further shows the association between CDAI and hyperlipidemia in male and female population. The results showed a statistically significant negative association between CDAI and hyperlipidemia in the female population after adjusting for potential confounders (OR = 0.98, 95% CI: 0.97–0.99). Even after dividing the study population into four groups according to CDAI, the risk of hyperlipidemia was 19% lower in the highest quartile compared to the lowest (OR = 0.81,95% CI:0.71–0.93). However, this association was not statistically significant in the male population (OR = 0.99, 95%CI: 0.98–1.00). In addition, the interaction showed that the negative association between CDAI and hyperlipidemia was more pronounced in the female population (P for interaction < 0.05).

**Table 3:** Association between CDAI and hyperlipidemia in different models among male and female.

	Crude model		Model 1		Model 2	
	OR (95%CI)	P	OR (95%CI)	P	OR (95%CI)	P
**Male**						
CDAI	0.98(0.97,0.99)	0.002	0.99(0.98,1.00)	0.016	0.99(0.98,1.00)	0.161
CDAI quartiles						
Q1	ref		ref		ref	
Q2	0.90(0.80,1.01)	0.079	0.84(0.75,0.95)	0.007	0.83(0.73,0.94)	0.003
Q3	0.87(0.76,1.00)	0.044	0.82(0.71,0.94)	0.005	0.81(0.70,0.94)	0.006
Q4	0.80(0.71,0.91)	<0.001	0.80(0.71,0.91)	<0.001	0.83(0.72,0.95)	0.006
*P for trend*		<0.001		0.002		0.014
**Female**						
CDAI	0.96(0.96,0.97)	<0.001	0.97(0.96,0.98)	<0.001	0.98(0.97,0.99)	<0.001
CDAI quartiles						
Q1	ref		ref		ref	
Q2	0.86(0.76,0.98)	0.026	0.85(0.74,0.97)	0.020	0.90(0.78,1.04)	0.159
Q3	0.85(0.74,0.98)	0.026	0.85(0.73,0.98)	0.029	0.94(0.81,1.10)	0.459
Q4	0.70(0.62,0.80)	<0.001	0.72(0.63,0.81)	<0.001	0.81(0.71,0.93)	0.003
*P for trend*		<0.001		<0.001		0.008
*P for interaction*	0.024		< 0.001		0.007	

Crude model: No adjusted.

Model 1: Adjusted for age, race, and PIR.

Model 2: Adjusted for age, race, smoking, drinking, education, type 2 DM, hypertension, marital status, PIR, BMI, CKD, and CVD.

Abbreviations: BMI, body mass index; PIR, poverty income ratio; DM, diabetes mellitus; CKD, chronic kidney disease; CVD, cardiovascular disease.

The interaction test is computed by treating CDAI as a continuous variable.

## Discussion

This study provides the first novel investigation of gender differences in the association between CDAI and hyperlipidemia using a large sample data set. After adjusting for potential confounders, the results suggest that higher levels of CDAI are associated with a lower prevalence of hyperlipidemia and that this association is nonlinear. In addition, we found that gender influenced the association between CDAI and hyperlipidemia, specifically that the association between CDAI and hyperlipidemia was more significant in female participants compared to the male population.

In recent years, the beneficial effects of CDAI on health have been extensively studied and confirmed to have protective effects against various diseases. Hu et al. conducted an observational study in postmenopausal women, revealing an association between CDAI and atherosclerotic, with a more pronounced effect observed in women aged 40–69, individuals with low HDL levels, and smokers [38]. Furthermore, CDAI has been linked to heart failure, stroke, and chronic obstructive pulmonary disease [29,39,40]. Multiple retrospective cohort studies in the US population have shown that CDAI is associated with reduced all-cause mortality and CVD mortality in stroke, CKD, type 2 DM, and cancer patients, consistent with our research findings, higher CDAI has been associated with a decreased prevalence of hyperlipidemia [41–43].

Furthermore, the association between dietary factors and hyperlipidemia has been widely discussed in previous studies, particularly focusing on the six dietary vitamins and minerals included in CDAI. In a randomized, double-blind study, supplementation of dietary vitamin C in hyperlipidemia patients significantly reduced lipid levels [44]. A cross-sectional study from the US found that higher dietary magnesium intake was associated with a reduced risk of hyperlipidemia [45]. Additionally, Huang et al. demonstrated lipid-lowering effects by supplementing hyperlipidemia mice with selenium-rich kiwifruit [46]. It was also found in another animal study that selenium supplementation improved dyslipidemia in rats [47]. Thus, dietary antioxidants play an important role in dyslipidemia.

In our study, we have provided the first evidence of a saturation effect between CDAI and hyperlipidemia. Specifically, we found that as CDAI increases, the risk of developing hyperlipidemia decreases rapidly. However, once CDAI reaches a certain inflection point, the rate of decline in the risk of hyperlipidemia becomes gradual. This finding is consistent with previous studies evaluating the relationship between CDAI and depression, as well as aging, it was emphasized that especially for people with low CDAI, there will be significant improvement through dietary supplementation with antioxidants [48,49]. In addition, we further found that the association between CDAI and the risk of developing hyperlipidemia was more pronounced in the female population by interaction examination, indicates that dietary supplementation with antioxidants is more effective for specific groups of women.

Although the mechanism underlying the association between CDAI and hyperlipidemia has not been extensively studied, the characteristics of CDAI suggest that this association may be explained by oxidative stress. A systematic review revealed that hypercholesterolemia can lead to oxidative stress by causing mitochondrial dysfunction [50]. In another study, Wen et al. demonstrated that effective antioxidants could reduce abnormal lipid metabolism in mice [51]. Furthermore, multiple studies have identified an association between lipid abnormalities and oxidative stress [52–54]. On a physiological level, excess reactive oxygen species (ROS) can lead to lipid peroxidation and consequently to the formation of oxidized low-density lipoprotein (Ox-LDL), a modified form of LDL with enhanced cytotoxicity and atherogenic properties [55]. In addition, oxidative stress may further exacerbate the inflammatory response and endothelial dysfunction by affecting key signal transduction pathways and transcription factors, such as the activation of nuclear factor kappa-B (NF-κ B) and mitogen-activated protein kinases (MAPKs), processes that are closely associated with dysfunctional lipid metabolism [56]. These findings help to explain the negative association between CDAI and hyperlipidemia.

In our study, we observed that the negative association between CDAI and hyperlipidemia was more significant in women, whereas it had little effect on men. This sex difference may be caused by several factors, including differences in hormone levels as well as lifestyle. Firstly, sex differences in physiology may have an impact on lipid metabolism and antioxidant status. Estrogen, in women, has been shown to influence lipoprotein metabolism, and estrogen may enhance endogenous antioxidant defense systems by upregulating the expression of antioxidant enzymes [57]. Secondly, lifestyle factors, including dietary habits, physical activity, and smoking and drinking behaviors, are potentially healthier in women, which may be associated with a higher CDAI [58]. Finally, socioeconomic factors may also play a role in sex differences. Women’s socioeconomic status may be more likely to influence their health awareness and nutritional choices, which may be reflected in CDAI scores [59].

Our study is the first to explore the association between CDAI and hyperlipidemia across genders and is based on data from a large sample. At the same time, we recognize several limitations of the study. Firstly, dietary data were obtained through recall of food intake over the past 24 hours, which may introduce recall bias. Secondly, our study design was cross-sectional, making it challenging to establish a causal relationship between CDAI and hyperlipidemia. Lastly, while the NHANES database facilitated our examination of the association between hyperlipidemia and CDAI, it is important to acknowledge that there are notable distinctions in the correlation between various clinical subtypes of hyperlipidemia and CVD as well as cerebrovascular diseases. Regrettably, our study did not delve deeper into analyzing the relationship between different clinical subtypes of hyperlipidemia and CDAI. By elucidating these nuanced distinctions, we could potentially propose more tailored and specific dietary intervention strategies for individuals with varying types of hyperlipidemias. This gap in our current research warrants further exploration and can be a valuable avenue for investigation in future cohort studies. It is important to consider these limitations when interpreting the conclusions of our research. Based on the limitations of our findings, we recommend that future studies use more detailed data and include a wider range of populations for randomised controlled studies.

## Conclusion

In this study, we found that higher CDAI was associated with a decreased risk of developing hyperlipidemia and that CDAI and hyperlipidemia showed an L-shaped relationship, not only that, this negative correlation is stronger in the female group. This result provides valuable insights for clinicians to enhance the health management of hyperlipidemia.

## Abbreviations

CDAI: composite dietary antioxidant index
BMI: body mass index
PIR: poverty income ratio
Type 2 DM: type 2 diabetes mellitus
CKD: chronic kidney disease
CVD: cardiovascular disease
HDL: high-density lipoprotein
LDL: low-density lipoprotein
RCS: restricted cubic splines
OR: odds ratio
CI: confidence intervals
NCHS: National Center for Health Statistics
NHANES: National Health and Nutritional Examination Survey
